# Supplementary figures and images for: A nonenzymatic dependency on inositol-requiring enzyme 1 controls cancer cell cycle progression and tumor growth
Source: PLoS Biol. 2025 Apr 10;23(4):e3003086. doi: 10.1371/journal.pbio.3003086 (PMC12080931; doi:10.1371/journal.pbio.3003086)

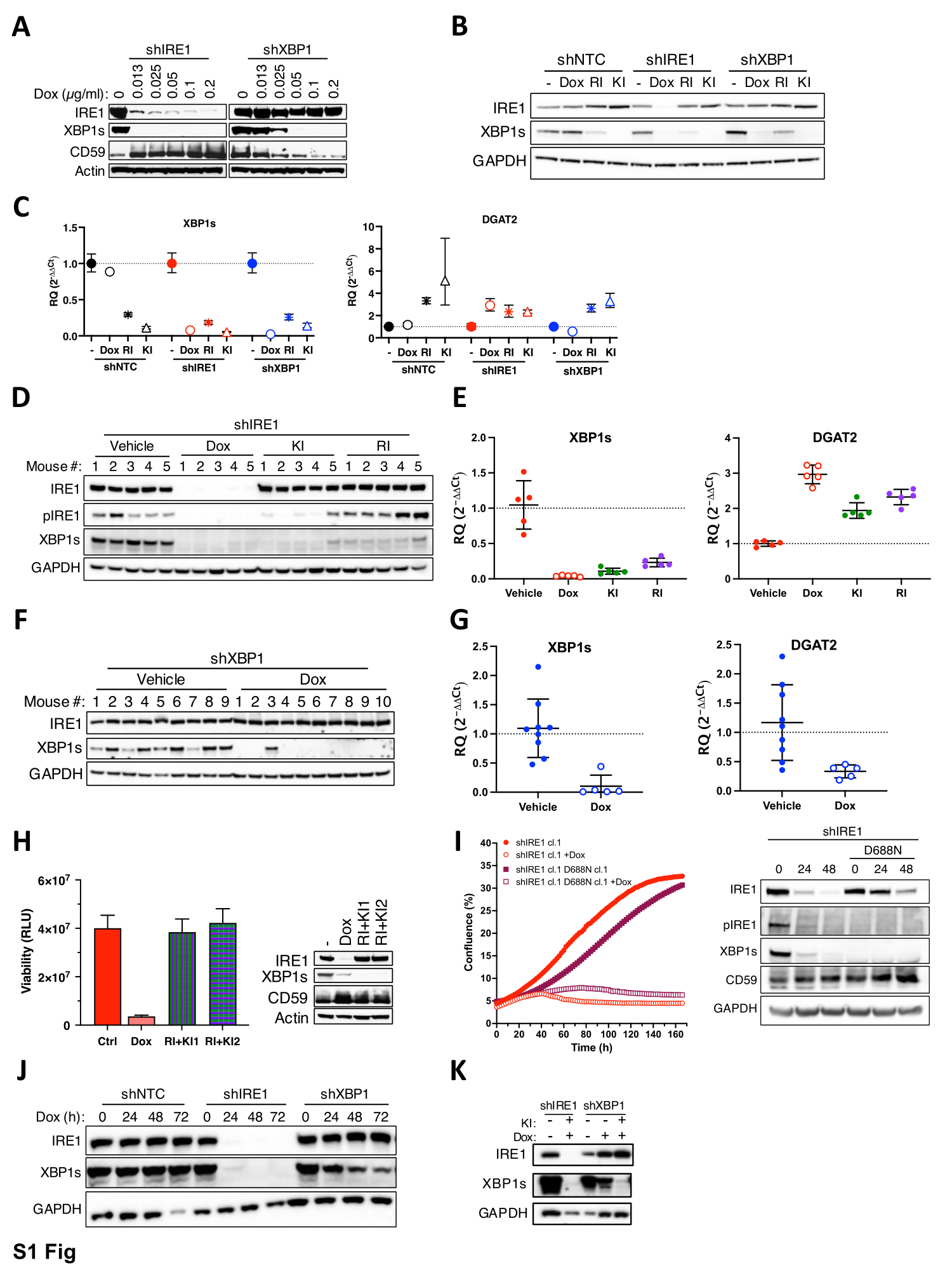

Supplement: S1 Fig — (A) Validation of IRE1 and XBP1s knockdown in AMO1 cells in vitro. Cells stably transfected with plasmids encoding Doxycycline (Dox)-inducible short hairpin RNAs (shRNAs) (3 distinct shRNAs in tandem) against either IRE1 or XBP1 were incubated for 48 h in the absence or presence of Dox at the indicated concentrations. Cells were then analyzed by Immunoblotting (IB). An independent clone shown for each gene (cl.1 for shIRE1 and cl.1 for shXBP1). (B) Validation of shRNA knockdown and enzymatic IRE1 inhibition in AMO1 cells. AMO1 shNTC, shIRE1 cl.1 and shXBP1 cl.1 cells were incubated for 48 h in the absence or presence of Dox (0.2 μg/ml), or IRE1 RI (3 μM), or IRE1 KI (KI2, 3 μM). Cells were analyzed by IB. Because XBP1s production requires both IRE1 kinase and RNase activity, its depletion confirms the inhibition of both functions. (C) Same cells as in B were analyzed by RT-qPCR for mRNA levels of XBP1s and DGAT2 (RIDD target). Data points are means of two technical replicates with error bars. Representative plot of three independent experiments shown. (D) Validation of shRNA knockdown and enzymatic IRE1 inhibition in AMO1 cells in vivo. C.B-17 SCID mice were implanted subcutaneously with 10 × 106 AMO1 shIRE1 cl.1 cells and allowed to form palpable tumors. Mice were then randomized into treatment groups (n = 5/group) and given either vehicle (5% sucrose) or Dox (0.5 mg/ml in 5% sucrose) ad libitum, or treated orally bidaily with vehicle, or IRE1 KI (250 mg/kg) or IRE1 RI (100 mg/kg) over 3.5 days. Tumors were collected 6h after the last oral dosing and analyzed by IB. (E) Mice were treated as in D and tumor samples were analyzed by RT-qPCR for XBP1s and DGAT2 mRNA levels as in C. Data points represent one biological replicate. Error bars represent SD. (F) Validation of XBP1 knockdown in vivo in AMO1 tumor xenografts. Tumors were collected at endpoint from the study depicted in Fig 1B. Tumor samples were analyzed by IB. (G) Tumor samples shown in F were analyzed as in [file pbio.3003086.s001.tiff]

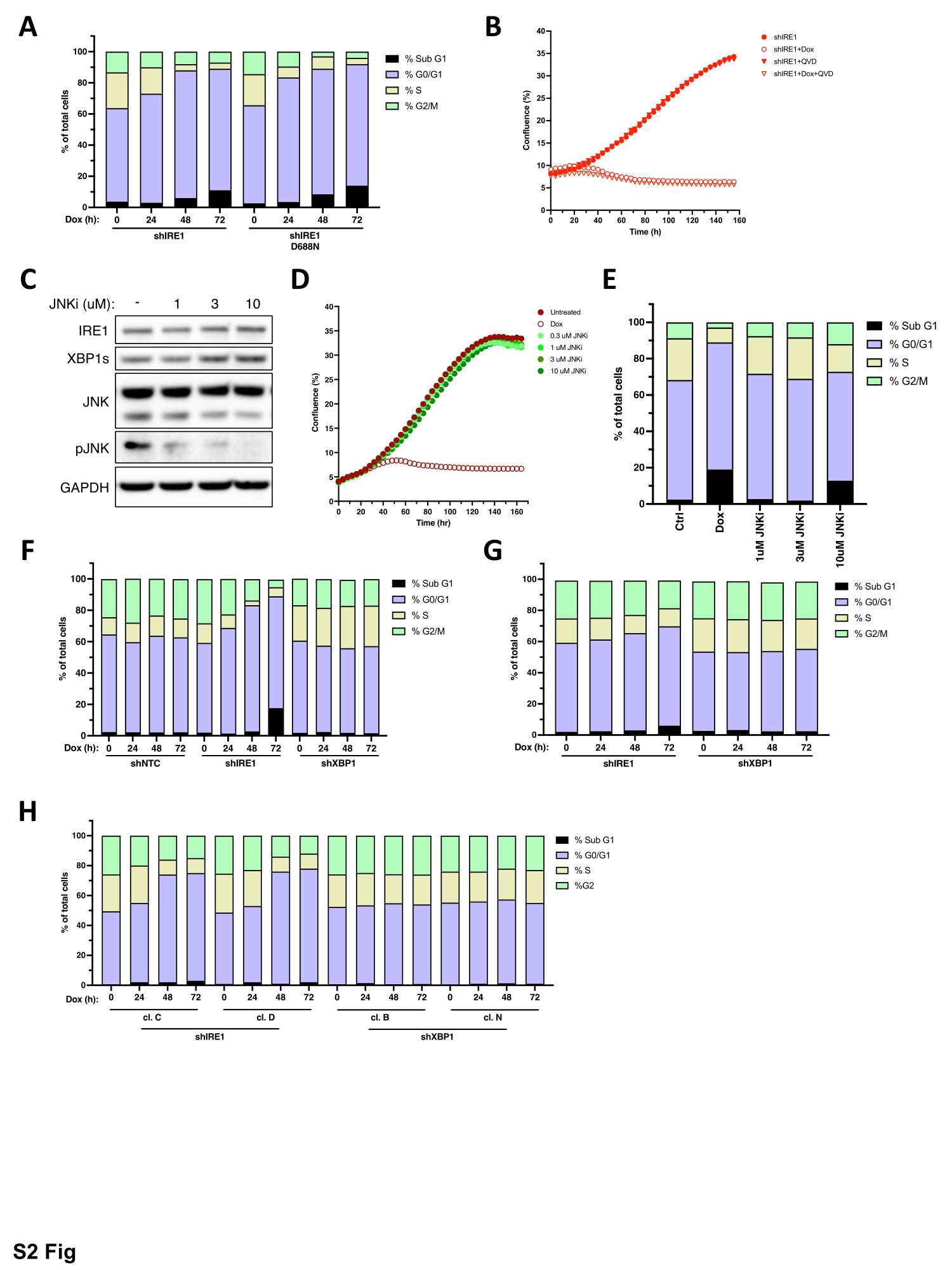

Supplement: S2 Fig — (A) AMO1 shIRE1 cl.1 or AMO1 shIRE1 kinase dead D688N mutant cl.1 cells were incubated with Dox (0.2 μg/ml) for the indicated time. Cells were stained with propidium iodide (PI) and analyzed by flow cytometry and cell frequencies by cell cycle phase were depicted as stacked bar graphs. Representative plot of 3 independent experiments shown. (B) Effect of QVD on AMO1 cell proliferation. AMO1 shIRE1 cl.1 cells were treated for 24 h with Dox (open symbols) (0.2 μg/ml) in the absence (circles) or presence (triangles) of QVD (30 μM) and analyzed for proliferation. Proliferation, depicted as % confluence, was monitored by time-lapse microscopy in an Incucyte instrument. Data points are mean of five technical replicates. Representative plot of 3 independent experiments shown. (C) AMO1 shIRE1 cl.1 cells were treated with JNK inhibitor Tanzisertib (JNKi) at the indicated concentrations for 48 h and analyzed for IRE1 and JNK pathway activation by IB. Representative blot of two independent experiments shown. (D) Effect of JNK inhibition on AMO1 cell proliferation. AMO1 shIRE1 cl.1 cells were incubated in the absence (closed symbols) or presence (open symbols) of Dox (0.2 μg/ml) or different concentrations of JNK inhibitor (JNKi, green). Proliferation was analyzed as in B. Data points are mean of five technical replicates. Representative plot of three independent experiments shown. (E) AMO1 shIRE1 cells were incubated with Dox (0.2 μg/ml) or different concentrations of JNK inhibitor (JNKi) for 48 h and analyzed as in A. (F) KMS27 shIRE1 cl.9 or shXBP1 cl.13 cells were incubated with Dox (0.2 μg/ml) for the indicated time and analyzed as in A. Representative plot of three independent experiments shown. (G) JJN3 shIRE1 cl.9 or shXBP1 cells were incubated with Dox (0.2 μg/ml) for the indicated time and analyzed as in A. Representative plot of three independent experiments shown. (H) L-363 shIRE1 or shXBP1 cells were incubated with Dox (0.2 μg/ml) for the indicated time. Two indepe [file pbio.3003086.s002.tiff]

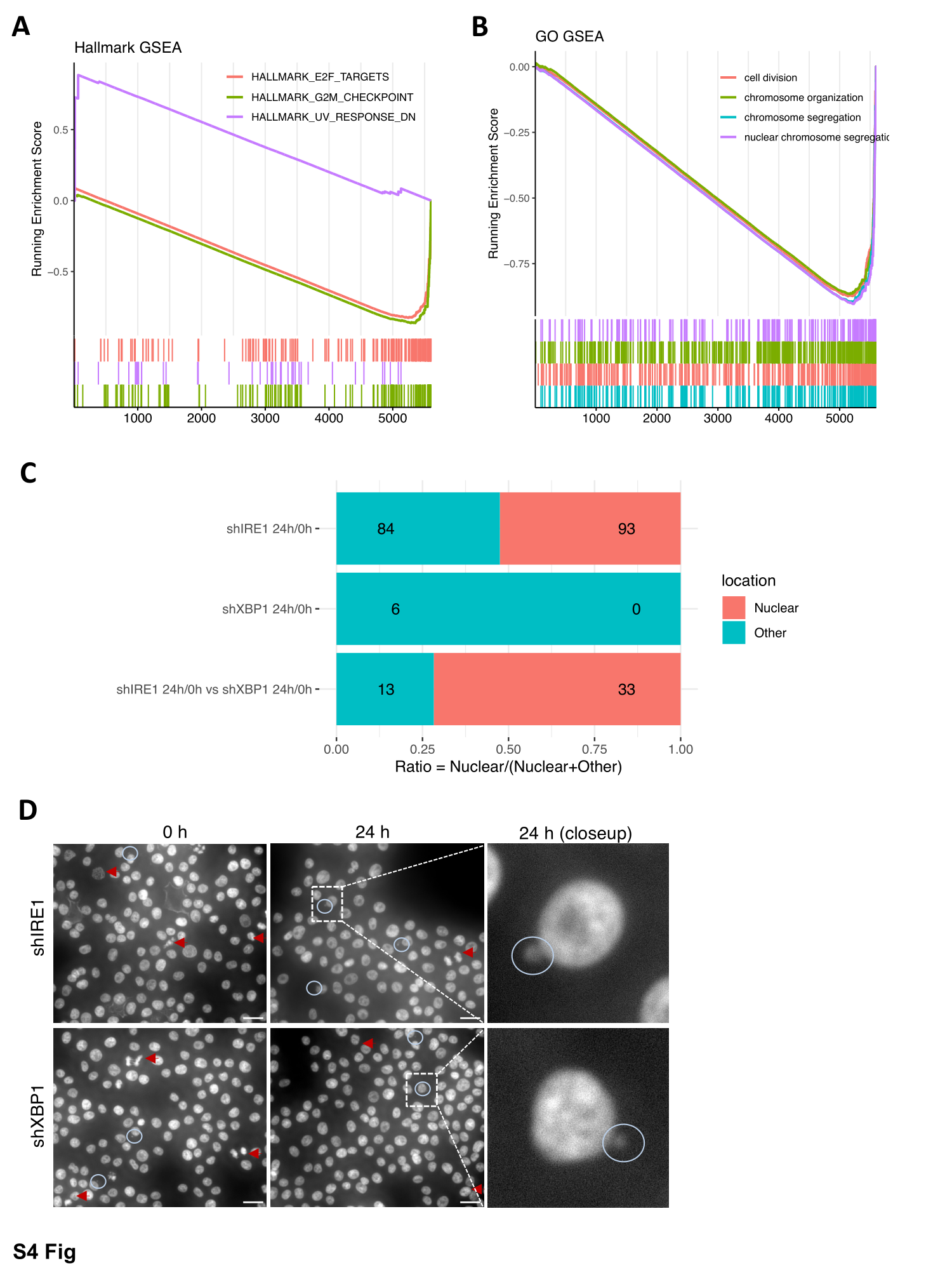

Supplement: S4 Fig — (A) Enrichment plots from the most significantly downregulated Hallmark gene sets corresponding to proteins regulated upon IRE1 knockdown versus XBP1 knockdown after 24 h. Data from the proteomics experiment shown in Fig 3F was used for the Hallmark GSEA. (B) Enrichment plots as in A for GO GSEA. (C) Effect of IRE1 or XBP1 knockdown on the fraction of nuclear versus non-nuclear proteins impacted based on proteomics results from Fig 3F. (D) Additional examples of cells analyzed as depicted in Fig 4A. Blue circles indicate examples of cells with micronuclei, while red arrows indicate mitotic cells. Scale bar = 20 μm. (E) Effect of IRE1 or XBP1 knockdown on frequency of micronuclei and mitotic events. KMS27 shIRE1 cl.9 and shXBP1 cl.6 were cultured in the presence or absence of Dox (0.2 μg/ml) for 24 h, stained with Hoechst DNA dye, and analyzed by fluorescence microscopy. Images representative of at least 10 fields examined per condition of three independent experiments. Blue circles indicate examples of cells with micronuclei, while red arrows indicate mitotic cells. Scale bar = 20 μm. (F) Quantification of annotated events in E normalized per total amount of cells per field. Ten fields per condition with at least 100 cells were counted for n = 3 independent experiments. Mean ± SEM of the fold change to untreated controls for each cell line. Mann–Whitney U statistical test. (G) Quantification of mitotic cells exemplified in E per total amount of cells per field. Ten fields per condition with at least 100 cells were counted for three independent experiments. Mean ± SEM of the fold change to untreated controls for each cell line. Mann–Whitney U statistical test. *p < 0.05; **p < 0.005; ***p < 0.001; ****p < 0.00001 consensus. A p value > 0.05 was considered non-significant (ns). All raw data can be found in S1 Data. (TIFF) [file pbio.3003086.s004.tiff]

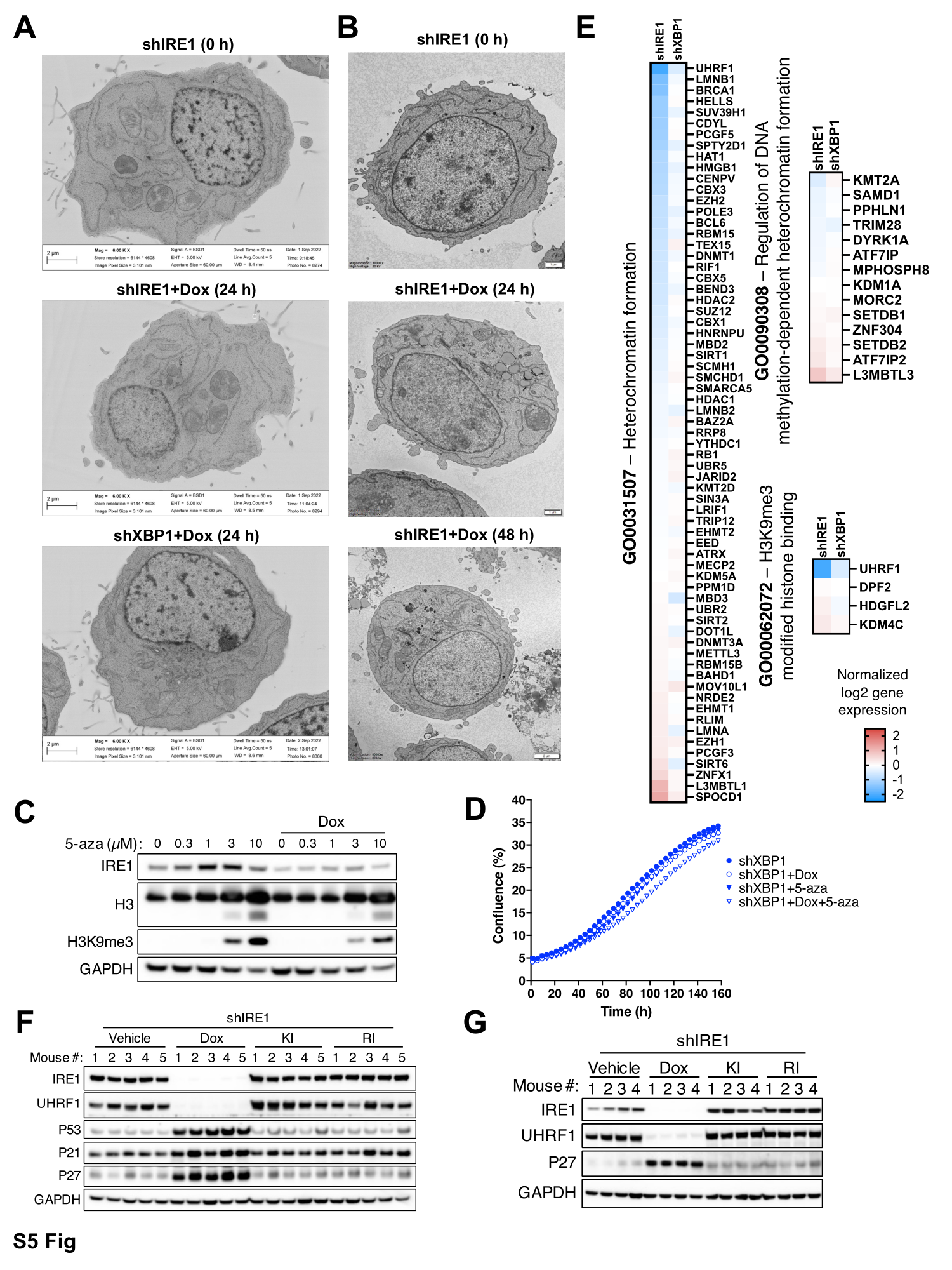

Supplement: S5 Fig — (A) Additional examples of cells analyzed as depicted in Fig 5A and 5B. (B) Examples of cells from an independent unbiased EM analysis performed at a different institution (Utrecht). AMO1 shIRE1 cl.1 cells were incubated for the indicated time with Dox (0.2 μg/ml). Scale bars are 1 μm. (C) AMO1 shIRE1 cl.1 cells were incubated for 24 h in the absence or presence of Dox (0.2 μg/ml) with the indicated concentration of 5-aza and analyzed by IB. The quantification for these IBs is depicted in Fig 5D. (D) Effect of XBP1 knockdown and 5-aza treatment on proliferation. AMO1 shXBP1 cl.1 cells were incubated for the indicated time in the absence (closed symbols) or presence (open symbols) of Dox (0.2 μg/ml), without (circles) or with (triangles) 5-aza (1 μM). Proliferation, depicted as % confluence, was monitored by time-lapse microscopy in an Incucyte instrument. Data points are means of five technical replicates. Representative plot of three independent experiments shown. (E) Effect of IRE1 or XBP1 knockdown on mRNA expression of heterochromatin formation and DNA methylation genes. Row-clustered heatmap depicting scaled mRNA expression by RNA sequencing of genes composing GO terms Heterochromatin Formation, Regulation of DNA Methylation-dependent Heterochromatin Formation, and H3K9me3 Modified Histone Binding for samples shown in Fig 3A. (F) Effect of IRE1 or XBP1 knockdown in vivo on UHRF1, p53, p21, and p27 protein levels. AMO1 tumor xenografts as depicted in S1D Fig were analyzed by IB. Note that the IRE1 and GAPDH blots from S1D Fig are duplicated here for direct reference. (G) KMS27 tumor xenografts as depicted in S1M Fig were analyzed by IB. Note that the IRE1 and GAPDH blots from S1M Fig are duplicated here for direct reference. All raw data can be found in S1 Data. (TIFF) [file pbio.3003086.s005.tiff]

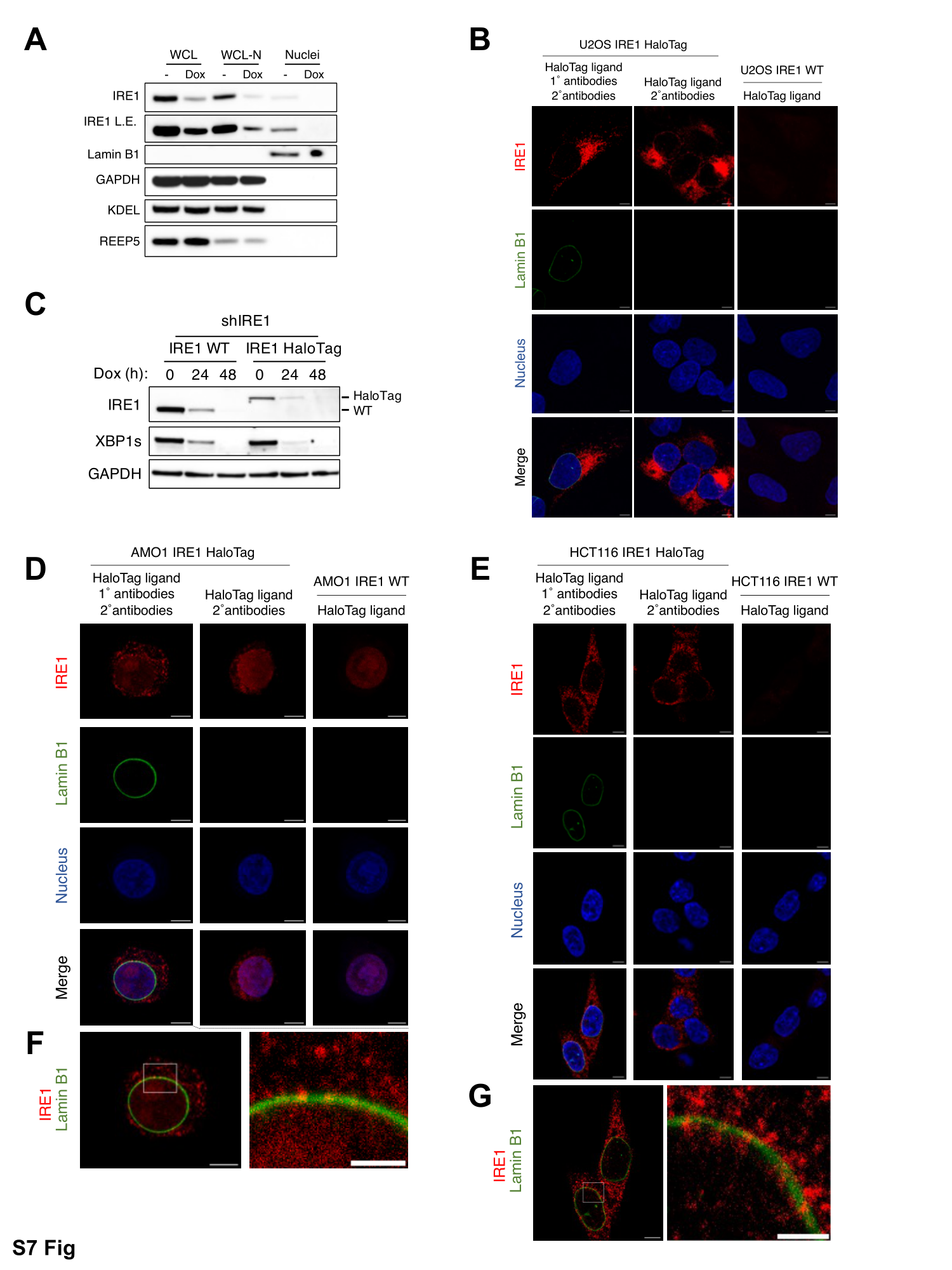

Supplement: S7 Fig — (A) AMO1 shIRE1 cl.1 cells were incubated in the presence or absence of Dox (0.2 μg/ml) for 24 h, subjected to subcellular fractionation, and analyzed by IB. WCL: whole-cell lysate. WCL-Nuc: Whole-cell lysate lacking the nuclear fraction. Nuc: nuclear fraction. The nuclear protein Lamin B1 and the ER markers KDEL and REEP5 were used to confirm the purity of the nuclear fraction. Representative blot of 2 independent experiments shown. (B) Additional controls of the experiment described in Fig 7B performed in both U2OS IRE1 HaloTag and U2OS wild type IRE1 (IRE1 WT) cells. Scale bar = 2 µm. Representative image of 2 independent experiments shown. (C) AMO1 shIRE1 cl.1 with wild type IRE1 (WT IRE1) or AMO1 shIRE1 cl.1 with endogenously tagged IRE1 (IRE1 HaloTag) cells were incubated for the indicated time in the presence of Dox (0.2 μg/ml) and analyzed by IB. Note that Halo-tagged IRE1 runs slower that WT IRE1 due to the increase in size produced by the HaloTag. Representative blot of three independent experiments shown. (D) and (E) Detection of endogenous Halo-tagged IRE1 and non-tagged Lamin B1 by confocal microscopy in AMO1 (D) and HCT116 (E) in both IRE1 Halo-tagged and WT cells. Cells were cultured in the presence of Janelia 646 HaloTag ligand that detects IRE1-HaloTag (red), fixed, stained with anti-Lamin-B1 (green) antibody, and analyzed by confocal microscopy. Individual and merge fields of IRE1 (red), Lamin B1 (green), and nucleus (blue) are shown. Scale bar = 5 µm. Pixel size = 48 nm for all images. Representative images of 2 independent experiments shown. Of note, AMO1 cells present nuclear autofluorescence in the far-red channel (HaloTag 646 channel) that doesn’t correspond to specific IRE1 staining as shown in the controls. (F) and (G) Left: Merge (left) and Close up (right) of the nuclear envelope for IRE1 and LaminB1 staining in AMO1 (F) and in HCT116 (G). Scale bar = 5 µm (merge) and 2 µm (close up). Pixel size = 48 nm for all images. (H) Additional examp [file pbio.3003086.s007.tiff]

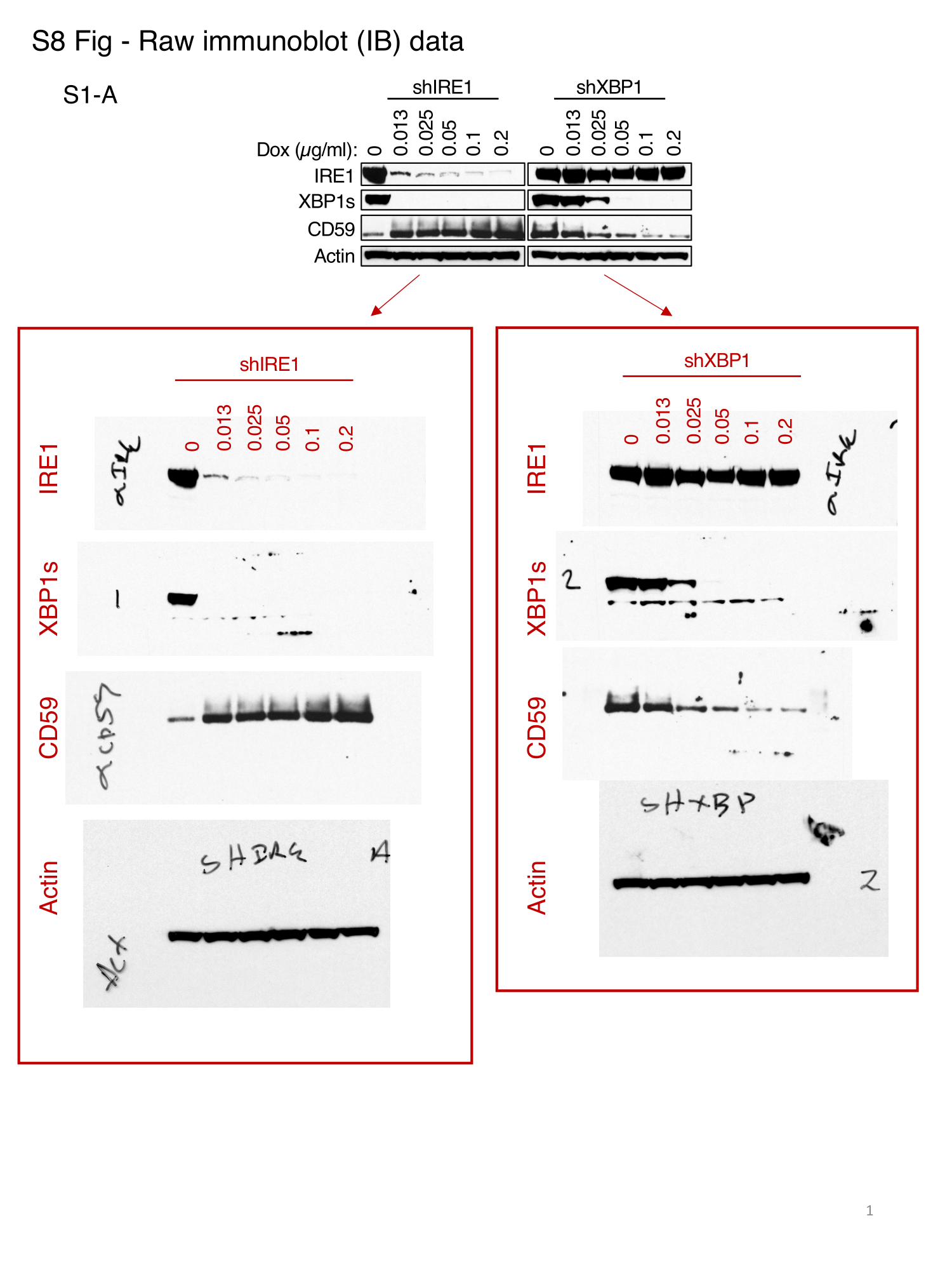

Supplement: S1 Raw Images — Each raw blot is labeled to annotate the loading order, experimental sample identity, protein blotted for and the figure that was generated from that original image. Lanes not included in the final figures are marked with an “X” above the lane label. (TIFF) [file pbio.3003086.s008.tiff]
