# Supplementary material for: A nonenzymatic dependency on inositol-requiring enzyme 1 controls cancer cell cycle progression and tumor growth
Source: PLoS Biol. 2025 Apr 10;23(4):e3003086. doi: 10.1371/journal.pbio.3003086 (PMC12080931; doi:10.1371/journal.pbio.3003086)
Supplement: Attachment — Submitted filename: PBIOLOGY-D-24-01245R2_ResponseReviewers.pdf [file pbio.3003086.s012.pdf]

PBIOLOGY-D-24-01245R1

Dear Dr Alvarez-Garcia,

Thank you for the invitation to revise the work to address the reviewers' concerts. We have performed several additional experiments as requested and believe we have fully addressed all of the reviewers' comments. Below please find a point-by-point response, which explains how we address each specific concern.

On behalf of the co-authors and myself, thank you again for considering our work for publication in PLOS Biology.

Sincerely,

Avi Ashkenazi, PhD

Dear Dr Ashkenazi,

Thank you for your patience while your manuscript entitled "A nonenzymatic dependency on inositol-requiring enzyme 1 controls cancer cell cycle progression and tumor growth" was peer-reviewed at PLOS Biology. Please also accept again my apologies for the delay in sending you our decision. The manuscript has now been evaluated by the PLOS Biology editors, an Academic Editor with relevant expertise, and by three independent reviewers.

The reviews are attached below. As you will see, the reviewers find the conclusions interesting and worth pursuing, but they also raise several concerns that should be addressed before we consider the manuscript for publication. Reviewer 1 notes that all the findings rely on shRNA knockdowns that have potential for off-target effects, thus this reviewer thinks that a rescue experiment using a shRNA-resistant IRE1 construct should be performed to validate that the main downstream effects are due to the absence of IRE1. This reviewer also finds the methods a bit confusing, and raises other issues that should be addressed. Reviewer 2 suggests several experiments to confirm the findings, such as analysing the role of the IRE1-TRAF2-JNK pathway in cancer development, and several missing controls, among other issues. Reviewer 3 thinks you should check if the inhibitors used have indirect effects in the cell culture models, and discuss if IRE1 could be overexpressed in cancer cells
to gain entry into the nucleus, acquiring new functions in cancer cells.

In light of the reviews and after discussion with the Academic Editor, we would like to invite you to revise the work to thoroughly address the reviewers' reports. Given the extent of revision needed, we cannot make a decision about publication until we have seen the revised manuscript and your response to the reviewers' comments. Your revised manuscript is likely to be sent for further evaluation by all or a subset of the reviewers.

We expect to receive your revised manuscript within 3 months. Please email us (plosbiology@plos.org) if you have any questions or concerns, or would like to request an extension.

At this stage, your manuscript remains formally under active consideration at our journal; please notify us by email if you do not intend to submit a revision so that we may withdraw it.

<u>Response to Reviewers Comments</u>

Rev. 1:
This study by the Ashkenazi group reports the discovery that some cancer cells are dependent on IRE1 but not on its enzymatic activities that mediate XBP1 activation or RIDD. In such cells, the absence of IRE1 results in cell cycle arrest, p53 activation, DNA damage, and chromosome instability. The main implications of this finding are two-fold. First, IRE1 has a clear non-enzymatic role in regulating cell cycle progression and DNA damage. Second, this alternative role is a potential therapeutic target in some cancers where simply targeting IRE1's enzymatic activities would not work.

The conclusion, if validated, would be a very important advance even if the molecular mechanisms of how IRE1 links to DNA damage and cell cycle progression are not clear yet. Thus, with a suitable validation of the non-enzymatic function of IRE1, I am strongly supportive of publication.

We thank the reviewer for provisionally recognizing the significance of our study and providing insightful and constructive suggestions for strengthening its conclusions.

Specific suggestions:

1) All of the downstream consequences of the putative non-enzymatic IRE1 function are well documented and clear. I therefore have no issues with any of this, although I admit that many of those areas are not my area of expertise. I am however concerned that all of the findings rely on shRNA knockdowns that have the potential for off-target effects. Thus, it seems critical to verify that the main downstream effects are due to the absence of IRE1. This is best accomplished by rescue experiments in which an shRNA-resistant IRE1 construct is used. In the absence of such a rescue, one would be concerned that the effects are due to perturbing some other gene product(s) whose absence is the basis of the observed phenotypes. I am not suggesting that everything be repeated with a rescue, but it does seem important to be certain that at least the key phenotypes are due to IRE1.

This is an important concern, and we agree that it is best addressed through a rescue experiment, which we have now performed. In short, we ectopically expressed shRNA-resistant BACs encoding either WT or mutant RNase-dead IRE1 (the latter being enzymatically inactive as demonstrated by its relative inability to recover XBP1 splicing). Importantly, as shown in new Fig. S1Y, compared to the empty vector both IRE1 variants provided a substantial and significant, albeit incomplete, rescue of viability in the context of Dox-induced IRE1 knockdown. (Note that potential limitations of BAC-mediated transient expression include the extra-genomic location of the plasmid and a possibility for mis-localization or misfolding of the encoded protein.) To further confirm the on-target nature of the observed phenotype, we depleted IRE1 using either an siRNA or an antisense oligonucleotide, each of which maps to a different region of the mRNA sequence (Fig. S1V). Both RNAi strategies clearly reduced cell viability in conjunction with clear depletion of IRE1 (New Fig. S1W and S1X). We further note that a non-targeting control shRNA was implemented in several of the original experiments (Figs. 1D, 1F, S1B, S1J, S1O), and that we employed Target Scan and BLAST methodology in our original shRNA design to maximize on-target selectivity. Taken together, these expanded results help validate the conclusion that the nonenzymatic dependency on IRE1 is an on-target phenotype.

2) The methods are slightly confusing to me. Does the BAC have all three shRNAs for IRE1, or are there three BACs each with a different shRNA? If the latter, did the authors sequence the shRNAs in the selected cell lines to know which one is actually being used? Right now, I cannot work out from the Methods which shRNA is actually responsible for the effects observed. Please clarify.

We apologize for not explaining this more clearly and have now added specific information to the Methods and Results sections to avoid ambiguity. In short, our Dox-inducible BACs against IRE1 or XBP1 each encode 3 tandem shRNAs, designed based on prior screening of multiple siRNAs by QPCR and immunoblot for optimal knockdown and on Target Scan (http://www.targetscan.org/vert_50/seedmatch.html) and BLAST for on-target selectivity.

3) The fact that some IRE1 can reside in the nuclear fraction is neither interesting nor illuminating. The ER is continuous with the outer NE, so unless a membrane protein has a preference for tubular ER and is unstable in flat membranes, it will be found in both the bulk ER and outer NE. The only situation where seeing nuclear IRE1 would be of interest is if it is in the inner NE and hence facing the nucleoplasm. If the authors are not able to show this, then I would eliminate the nuclear localization observation (or at least not make such a big deal of it).

We agree that the localization of an ER-transmembrane protein such as IRE1 to the outer nuclear envelope (NE) is not surprising and we have toned back our comments in this regard. Nevertheless, while being a reasonable theoretical expectation, we believe it is important to demonstrate it empirically. Indeed, it is conceivable that unknown tethering or restriction mechanisms could prevent IRE1 from distributing to the NE. Furthermore, by establishing this first available immuno-EM methodology to study the *endogenous* IRE1 protein, we are paving a path for future investigations of its heretofore poorly defined role in the NE. Incidentally, reviewer #3 asked for further validation of the localization, which now provide in Figs. 7 and S7.

Rev. 2:
This study investigated the role of endoplasmic-reticulum resident inositol-requiring enzyme 1α (IRE1) in cancer cell progression independent of its enzymatic activity. Knockdown of IRE1 expression attenuated cancer cell cycle and tumor growth, unlike enzymatic IRE1 inhibition or XBP1 knockdown. Mechanistically, IRE1 knockdown increased TP53 and CDKN1A/p21 expression, promoted DNA and H3 methylation, chromosome instability, and heterochromatin formation. Additionally, IRE1 was found to localize to the nuclear envelope. It is concluded that IRE1a regulates cancer cell progression independent of its enzymatic activation.

General Comments:
The study suggests a nonenzymatic role for IRE1 in cancer cell development, but there are significant issues with over-conclusions and experimental reliability.

We appreciate the reviewer's diligence and constructive comments for strengthening the conclusions.

Specific Comments:

1. IRE1-specific Inhibitors: The study uses IRE1-specific inhibitors, but these can affect other pathways. Constructing vectors for IRE1 enzyme inactivation would better verify its function.

We assume that the phrase "vectors for IRE1 enzyme inactivation" refers to a mutational disruption of IRE1's enzymatic activity. To this end, given that IRE1's kinase activity is required for its RNase activation, we disrupted the kinase via CRISPR/CAS9-mediated homozygous D688N mutation of the endogenous IRE1 (ERN1) gene locus in AMO1 cells, thus generating a kinase-dead IRE1 mutant. As expected, this mutation abolished both IRE1 phosphorylation and XBP1 splicing, confirming that it disabled both the kinase and RNase activity of IRE1 (Fig. S1I). Importantly, cells expressing D688N proliferated similarly to cells expressing WT IRE1, while Dox-induced shRNA silencing of IRE1 in either context still blocked proliferation (Fig. S1I) and promoted cell cycle arrest (Fig. S2A). Also relevant here is new data provided in response to Reviewer #1's first comment, which shows that ectopic expression of shRNA-resistant WT IRE1 or RNase-dead mutant IRE1 significantly rescued loss of viability due to knockdown of endogenous IRE1 (Fig. S1Y).

2. Contradictory Results with XBP1: Figures 2B and 2C show that XBP1 silencing or enzymatic IRE1 inhibition does not affect the cell cycle, contradicting previous findings that XBP1 regulates cell cycle genes. The authors should further investigate this discrepancy.

Previous reports in the literature already indicated that cell cycle regulation by XBP1 is not uniform. For example, Gomez et al. (DOI: 10.1096/fj.06-7990com) showed that overexpression of XBP1s in MCF7 cells increased the proliferative fraction of cells in S-phase, while Spaan et al. (DOI: 10.1038/s41419-019-1729-4) showed that XBP1s overexpression induced a G1 arrest in LS174T cells. We already included data as an example of a cell line (OPM2) that displays comparable dependency on IRE1 and XBP1 for cell growth (Fig. S1T-U) and cell cycle progression (Fig. S2O), illustrating enzymatic dependency on IRE1. To expand on this, we now provide additional data for the KMS11 cell line (Fig. S2P), which also displays a comparable cell-cycle dependency on IRE1 and XBP1. Further validation of such dependency can be found in Harnoss et al. PNAS 2019 (DOI: 10.1073/pnas.1906999116). Thus, both published data and results herein indicate that cell cycle regulation by XBP1s is context dependent, rather than contradictory.

3. Mechanism of IRE1 Silencing: The study shows IRE1 silencing downregulates many cell cycle genes independently of XBP1. The regulatory mechanism needs further clarification, including results from IRE1 enzyme inactivation.

As noted in the text, this question is more appropriate for future studies. For a further perspective on this point, please refer to reviewer #1's statement that "the findings represent a very important advance even if the molecular mechanisms of how IRE1 links to DNA damage and cell cycle progression are not clear yet." We also note that a follow-up manuscript, focused entirely on the underlying mechanism, is currently under review at PLOS Biology.

4. Expression of p53 and p21: Figure 3H shows decreased p53 and increased p21 with shIRE1. The authors need to explain this unexpected result.

The figure actually shows an initial increase in p53 from 6 to 24 hr followed by a decline at 48 and 72 hr, while p21 upregulation begins at 12 hr and reaches a plateau between 24

and 72 hr. It is known that the p53 and p21 proteins are controlled by distinct regulatory mechanisms. For example, p53 protein is directly controlled by the E3 ubiquitin ligase MDM2, while p21 protein is not. Accordingly, one might expect them to show different kinetic profiles after induction. An additional factor that can specifically affect p53 is caspase-mediated cleavage, as reported by Sayan et al. (DOI: 10.1074/jbc.M512467200). Indeed, as we now show in Fig. S3H, addition of the pan caspase inhibitor QVD stabilized the upregulation of p53 upon IRE1 knockdown at least through 48 hr.

5. IRE1-TRAF2-JNK Pathway: The study should examine the IRE1-TRAF2-JNK pathway's role in cancer development to support the hypothesis.

We appreciate this interesting suggestion. To investigate whether the JNK pathway is involved, we leveraged the highly selective JNK inhibitor Tanzisertib (CC-930), described by Plantevin-Krenitsky et al. (DOI: 10.1016/j.bmcl.2011.12.027). Our new data shows that Tanzisertib effectively blocks JNK phosphorylation upon addition at 1 - 10 uM to AMO-1 cells (Fig. S2C). Importantly, JNK inhibition had no effect on proliferation or cell cycle progression (Fig. S2D and E). These results exclude the JNK pathway as a key mediator of nonenzymatic dependency on IRE1.

6. Micronuclei Frequency: Figure 4B shows significant micronuclei increase with IRE1 silencing, but Figure 4A does not. This experiment should be repeated in other cancer cell lines.

Fig. 4B shows data collected from 30 fields per condition, while Fig. 4A shows a single, representative field and a higher magnification of a section thereof, for each experimental set. Note that in Fig. 4A for shIRE1, the 0-hr image has a single micronucleus, while the 24-hr image has 2 micronuclei, which is consistent with the 2-fold increase seen in Fig. 4B. Moreover, both the 0-hr and 24-hr images for shXBP1 each show a single micronucleus, again consistent with the data seen in Fig. 4B. To further illustrate the effect on micronuclei, we have now included an additional set of images in Fig. S4D. Furthermore, as requested, we performed similar experiments in KMS27 cells and the data is now included in Fig. S4 E-G.

7. Statistical Significance: Figures 4B, 4C, 4D, and 4E lack p-values and should include them for clarity.

We apologize for this oversight. The definition of the asterisk is now included in the legends.

8. Choice of UHRF1 Gene: The rationale for choosing UHRF1 over other genes involved in DNA methylation and chromatin modification should be explained. The RNA-seq results should also include DNA demethylation genes.

We appreciate this helpful recommendation. To clarify the rationale for focusing on UHRF1, we have re-ordered the panels in Fig. 5. It now begins with the data on the loss of heterochromatin (Fig. 5 A,B and Fig. S5 A,B). We then show changes in DNA and histone H3 methylation (Fig. 5C and D). This provides the motivation for investigating factors that regulate heterochromatin formation. We then include—as suggested by the reviewer—new analyses of the RNA-seq results (shown as heatmaps), focused on genes involved in regulating heterochromatin formation, DNA methylation-dependent heterochromatin formation, and H3K9me3-modified histone binding (Fig. S5E). Amongst the first and third

gene sets UHRF1 showed the most notable downregulation in response to IRE1 knockdown, explaining why we chose to further analyze it, as now shown in Fig. 5G.

9. Cyclin Protein Expression: To prove IRE1's independent regulation of cyclin protein expression, add a kinase mutant group and ShRNA-XBP1 group in Figures 6 and S6.

We thank the reviewer for this suggestion. We performed additional immunoblot analyses of several cell cycle markers in cells expressing the kinase-dead D688N IRE1 mutant and of cells expressing shXBP1; we also included the kinase and RNase inhibitors of IRE1 (blots added to Fig. S6A and B). The results confirm the nonenzymatic dependence on IRE1.

10. Immunofluorescence Clarity: Figure 7B immunofluorescence results are unclear and lack a scale. The experiment should be repeated in other cancer cell lines.

We appreciate this feedback. We have repeated the immunofluorescence studies and now provide clearer images in Fig. 7B (with controls in Fig. S7B). Furthermore, we have added immunofluorescence data for AMO-1 and HCT116 cells Fig. (S7D-G).

11. Nuclear Localization of IRE1: The study claims IRE1 localizes in the nucleus, but the mechanism and its relation to enzyme activity and cell cycle regulation need clarification.

As discussed above in connection with comment #3, we believe this mechanism would be beyond the scope of the present study and more appropriate for future investigations. Reviewer #1 agrees that the present study provides a very important advance even if the molecular mechanisms underlying the phenotype are not clear yet.

Rev. 3:
Zuazo-Gaztelu et al. describe a non-enzymatic function of IRE1 in regulating the cell cycle in certain cancer cells. IRE1 is an endoplasmic reticulum (ER) localized transmembrane kinase and RNase. Upon ER stress, IRE1 activates the XBP1 transcription factor, which upregulates many genes responsible for mitigating ER stress. IRE1 can also directly cleave certain mRNAs and miRNAs, which are implicated in reducing ER load and inducing cell death. In this manuscript, the authors have devised an elegant approach to deplete IRE1 using acutely shRNA in various cancer cell lines. They found that acute depletion of IRE1 resulted in inhibition of cell cycle progression in cancer cells in both in vitro and in vivo. By performing RNA seq, proteomics, and imaging, the authors observed the activation of the p53 pathway, DNA damage, and chromosome instability in some cancer cells. Therefore, the authors conclude that IRE1 functions as a scaffold to regulate cell cycle progression via unknown factors. Overall, this manuscript provides a new function of IRE1 and opens a new way to target IRE1 for therapeutic benefits. I suggest that the authors address the following concerns.

We thank the reviewer for kindly recognizing the elegance of the approach used to acutely disrupt IRE1 via inducible shRNA silencing and for acknowledging that the findings provide a new function of IRE1 and opens a new way to target IRE1 for therapeutic benefits.

1. The use of IRE1 kinase and RNase inhibitors suggests IRE1's non-enzymatic function. Inhibitors could have indirect effects. The authors support their core findings by complementing IRE1 kinase and RNase mutants into IRE1-depleted cells. This can be done only in cell culture models and does not necessarily need to be shown with animal models.

We share the reviewer's general reservation about selectivity of enzymatic inhibitors. However, please note that this would be more of a concern if the IRE1 inhibitors produced a phenotype whereas genetic IRE1 disruption did not. In the present case, the converse is true. Nevertheless, we appreciate the suggestion to use enzymatic mutants to provide further validation. We have now included new data showing that kinase-dead IRE1 still supports proliferation and cell cycle progression (Fig. S1I and S2A), and that ectopic expression of shRNA-resistant cDNA encoding either WT or RNase-dead IRE1 significantly rescues cell viability in the context of endogenous IRE1 knockdown (Fig. S1Y).

2. It is difficult to rationalize that a low-abundant protein, IRE1 (~416 molecules/HeLa), functions as a scaffold protein that regulates the cell cycle (PMID: 24487582). Also, most endogenous IRE1 is bound to the Sec61 translocon in cells (PMID: 25993558). It is possible that IRE1 is overexpressed in cancer cells to gain entry into the nucleus, thus acquiring new functions in cancer cells. The authors should discuss this.

This is an interesting consideration. Based on the Broad Institute's DepMap database, the levels of IRE1 can markedly vary between cancer cell lines (graphed below, with several relevant cell lines annotated). Indeed, HeLa cells have among the lowest levels of IRE1. However, while AMO1 and KMS27 have relatively high levels, HCT116 does not, so there is no clear correlation between IRE1 expression levels and non-enzymatic dependency on IRE1. We added IRE1 expression data from DepMap to Fig. S7I and note this in the Discussion.

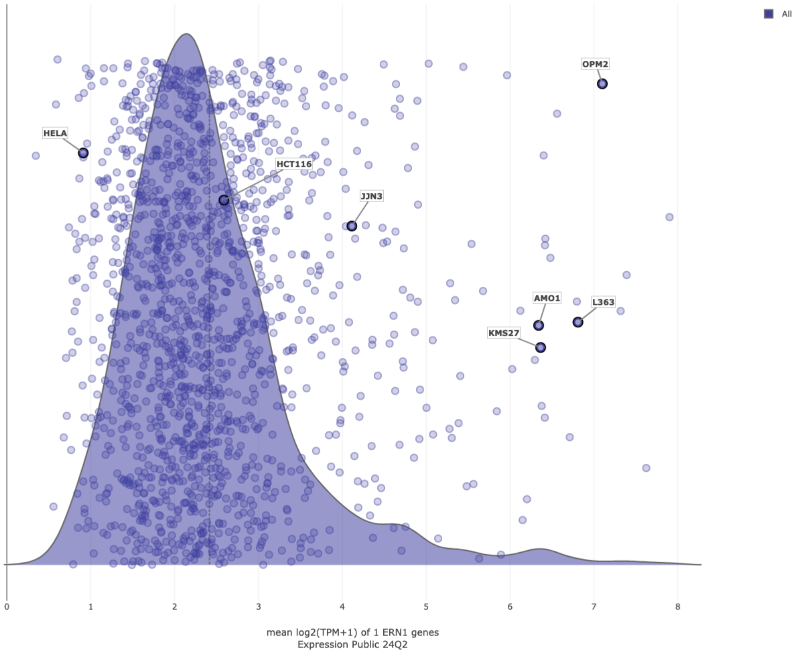

Minor concerns:
1. The resolution of the main figures is very poor.
This issue will be taken care of in the final version of the manuscript.

2. The scale bar is missing in Figures 4A and 7B.
We apologize for this oversight: scale bars are now added.

3. It is unclear why XBP1s is noticeably detected only upon Tg treatment in Fig. S1O, whereas it is detected without ER stress in other figures.

Unlike the other cell lines we studied, the JJN3 cell line shows low baseline levels of XBP1s. Therefore, to better visualize the effect of Dox-induced shRNA knockdown of IRE1 or XBP1 on XBP1s levels, we treated the cells with Tg to induce better detectable levels of the protein. The data shows that either IRE1 or XBP1 shRNA block XBP1s induction by Tg. This is now explained in the legend.
